# Ossification of the Placenta

**Published:** 1874-01

**Authors:** A. G. Smythe

**Affiliations:** Gun Town, Mississippi


### BREVITIES.

Ossification of the Placenta.—Mrs. Elizabeth E., aged 18, prim., heavy-set and rather corpulent, was seized with active symptoms of labor at the end of the seventh month of gestation. The pains continued at short and regular intervals for twenty-four hours, when violent signs set in. The pains were relieved only by the use of opium in some form, and the rigors could only be controlled by the free inhalation of chloroform. Pains and rigors continued about the same way for ten days. Relieved by the opium and chloroform, after which they returned at intervals of seven or eight days, being relieved as before. True labor commenced at 1 P.M., the 21 August, precisely forty-six days after the first attack, and was terminated in thirteen hours moderate labor, by the birth of a moderate sized male child. The placenta had to be detached, in doing which I discovered there was an unusual feeling, very much like the feeling of freshly cut beef in coarse salt. Upon a close examination, the whole of the attaching surface of the placenta was covered with a kind of net-work of bone, in many places there were solid plates as large as a dime coin, and a line in thickness. There was nothing more unusual in the size or appearance of the placenta.

Mrs. E. made a rapid recovery with only one symptom of the rigors at the end of the twenty-first day, which disappeared so soon as the chloroform was used.

The rigors had none of the symptoms of puerperal convulsion, they were purely paroxysms of violent shivering unaccompanied with anything like spasm, convulsion on any sensation of cold; the temperature of the air was high the greater part of the time during the confinement, and the patient suffered much from the excessive warm weather. A. G. Smythe, M.D.

Gun Town, Mississippi.

